# Compliance with Tuberculosis Screening in Irregular Immigrants

**DOI:** 10.3390/ijerph16010028

**Published:** 2018-12-23

**Authors:** Francesca Bonvicini, Silvia Cilloni, Rossano Fornaciari, Carmen Casoni, Cristina Marchesi, Marina Greci, Lucia Monici, Fausto Nicolini, Marco Vinceti

**Affiliations:** 1 Azienda Unità Sanitaria Locale di Reggio Emilia, Reggio Emilia 42122, Italy; francesca.bonvicini2@ausl.re.it (F.B.); rossano.fornaciari@ausl.re.it (R.F.); carmen.casoni@ausl.re.it (C.C.); cristina.marchesi@ausl.re.it (C.M.); marina.greci@ausl.re.it (M.G.); lucia.monici@ausl.re.it (L.M.); fausto.nicolini@ausl.re.it (F.N.); 2 Università degli Studi di Modena e Reggio Emilia, Dipartimento di Scienze Biomediche, Metaboliche e Neuroscienze, Modena 41125, Italy; silvia.cilloni@unimore.it

**Keywords:** tuberculosis, screening for tuberculosis, public health, immigrants

## Abstract

Tuberculosis (TB) is a serious public health problem in many regions of the world, especially in the poorest areas. For this reason, screening for active and latent forms must be considered when dealing with high-risk groups such as irregular immigrants in Western countries. We conducted a retrospective cohort study by recruiting subjects aged ≥15 years who underwent a tuberculin skin test at a dedicated National Health Service Centre in a northern Italian province between 1 January 2012 and 31 December 2013. These participants were followed up until 31 December 2016. We aimed at evaluating an experimental protocol for active and latent tuberculosis screening, focusing on patient compliance, feasibility, and capability to detect clinical forms of the disease. We enrolled 368 irregular immigrants, i.e., immigrants not having a valid residence permit and who were therefore not entitled to choose a general practitioner. In total, 90.22% of these completed all the steps for the screening of active TB, while 87.33% also undertook screening for the latent form of the disease. Homelessness, self-reported prostitution, female sex, and employment status adversely affected compliance. Chronic alcohol consumption was associated with increased risk of no beginning or interruption of the procedures. All of the five patients with active TB successfully completed the treatment. Overall, adherence to the screening program was high compared to other studies in immigrants, possibly owing to organizational factors such as the availability of cultural mediators, the network between the different health services, the presence of dedicated nursing staff and a free-of-charge service. In addition, selected vulnerable subgroups should be targeted using tailored screening and follow-up programs.

## 1. Introduction

Tuberculosis (TB) is the main cause of death from infectious disease globally. Drug-resistant forms of the disease are a major risk to global health security [1]. The WHO estimates that 10.4 million individuals became ill with TB and 1.7 million died in 2016. Despite a fall in mortality rate by 3% per year, TB remains the ninth leading cause of death worldwide, resulting in 1.3 million deaths in HIV-negative people and almost 400,000 deaths in HIV-positive people [2,3]. TB epidemiology in most low-incidence countries is characterized by a low rate of transmission in the general population, occasional outbreaks and a majority of TB cases generated from progression of latent tuberculosis infection (LTBI) rather than local transmission [4]. Migration flows have changed drastically since the beginning of the 21st century. Because most immigrants come from countries with a high incidence of tuberculosis, the contribution of the migrant population to new cases of tuberculosis is stronger in relative terms than for its weight in the total population [5]. For this reason, it is necessary to both diagnose TB early by including universal drug susceptibility testing, and to implement systematic screening for TB in selected high-risk groups. Early detection helps to reduce the risk of further TB transmission, poor treatment outcomes and undesirable health sequelae, thus also decreasing adverse social and economic consequences of the disease [6,7].

In the last 50 years, the annual incidence of TB in Italy decreased by about 70%, from around 25 to 7 cases per 100,000 individuals, and it now seems to be quite stable [8]. In this country, however, only limited data are available about TB epidemiology in potentially high-risk groups such as undocumented immigrants. Consequently, TB prevalence and risk factors in illegal immigrants are unknown. Furthermore, they have not been monitored over time. In spite of this, while national guidelines [9] recommend LTBI screening in high-risk subjects such as pulmonary TB contacts, HIV-infected patients, and patients undergoing immunosuppressive therapy and health care workers, less attention is devoted to the immigrant population, whereby early detection of active and LTBI cases should be pursued using the following strategies [10]: screening for both active TB and LTBI and therapy of LTBI in children, chest X-ray (CXR) screening for active TB in symptomatic subjects especially recent irregular immigrants. In addition, screening for active and latent TB in asymptomatic adults with recent immigration (<5 years) or living in social and health conditions of marginalization has no strong evidence. This also applies to undocumented immigrants, although these may represent an important source of LTBI and active TB. At the Centre for Health of Foreign Family (CFF), systematic screening for both active TB and LTBI had been performed since January 2012.

## 2. Materials and Methods

### 2.1. Setting and Clinical Procedures

The study protocol was approved by the Ethics Committee of the Reggio Emilia Province on 19 April 2017 and by the Reggio Emilia Local Health Unit on 5 June 2017 (document code 2017/DS/0038). 

This study took place in the CFF of Reggio Emilia, an area in northern Italy with approximately 550,000 inhabitants. The CFF is an outpatient clinic located in the city centre. It is devoted to immigrants without a valid residence permit, who are not entitled to choose a general practitioner. 

**Figure 1:**
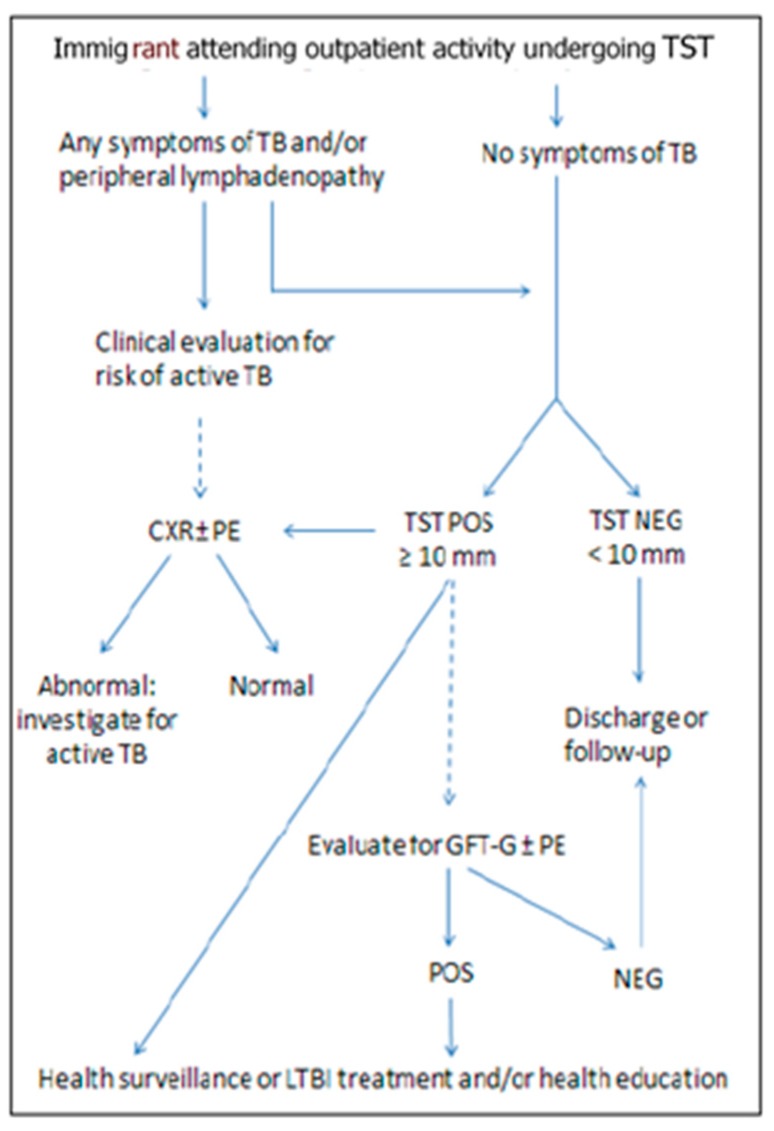
Procedures for active tuberculosis (**TB**) and latent tuberculosis infection (**LTBI**) screening at the Centre for Health of Foreign Family (**CFF**) of Reggio Emilia. **TST**: Tuberculin Skin Test; **CXR:** chest X-ray; **PE:** Pneumological Examination; **POS:** positive; **NEG:** negative.

In accordance with national recommendations [10], the reference test for the diagnosis of LTBI is the tuberculin skin test (TST) based on the Mantoux method. TST is carried out by the nursing staff in accordance with the Mantoux method, by inoculating 0.1 mL of purified protein [11]. The nursing or medical staff read the induration in millimetres after 48–72 h. If positive, the TST is always evaluated by medical personnel. The cut-off for TST positivity is usually 10 mm. In case of immunosuppression, it drops to 5 mm. Screening is performed according to a local protocol based on the combined use of TST and interferon gamma release assay (IGRA). Specifically we have used Quantiferon-TB Gold (QFT) for evaluating the possible presence of active TB. In Figure 1 it is possible to see the algorithm used at CFF for the screening of active TB and LTBI; in immigrants with any symptom of TB and/or peripheral lymphadenopathy, TST is performed as a first step. As far as TB symptoms are concerned, we consider at least one of the following: cough, haemoptysis, fever, chest pain, weight loss, fatigue, night sweats, chills or loss of appetite. The clinician visiting the patient may prescribe CXR at the time of TST and at any time during the screening protocol if a high risk for active TB is estimated. If the CXR shows any abnormality, the patient is referred for the pneumological examination (PE) or hospitalization. If CXR is negative, the patient’s LBTI diagnosis can be proceeded with. TST-positive patients undergo CXR (if not yet performed) and possibly QFT. QFT is generally prescribed for confirmation for the purpose of offering LTBI treatment. The indication for LTBI treatment is decided by the pneumologist, after patient examination and evaluation of CXR and QFT results. For LTBI treatment, we generally prescribe isoniazid for 6 months at a dose of 5 mg/kg/day, combined with vitamin B6, in order to prevent neurological adverse effects. Isoniazid preventive therapy (IPT) is recommended in people aged ≤35 years and in people of any age with HIV co-infection. In case of contraindication, failure to accept or need to discontinue IPT, patients with LTBI undergo health surveillance (HS) through clinical examination every six months for two years, for the purpose of early detection of the onset of active TB symptoms.

All examinations and visits are free of charge. Cultural mediators belonging to the most common ethnic groups of patients are constantly present to assist health personnel and immigrants during outpatient activity.

### 2.2. Study Design and Definitions

In this retrospective cohort study, all foreign-born patients aged ≥15 years who underwent TST at the CFF between 1 January 2012 and 31 December 2013 were enrolled, provided that there was no evidence of previous contact with a case of pulmonary TB or positive TST. The period of active follow-up in patients undergoing HS ended on 31 December 2016. We defined patients with LTBI as immigrants with both positive TST and QFT and patients with positive TST but QFT not requested or not performed. We considered patients without LTBI as subjects with negative TST and with positive TST but negative QFT. According to the World Health Organization [12], a bacteriologically confirmed TB case was one from whom a biological specimen was positive by smear microscopy, culture or rapid diagnostic tests (such as Xpert MTB/RIF assay that is a nucleic acid amplification test). A clinically diagnosed TB case was one diagnosed with active TB by a clinician or other medical practitioner who decided to start a full course of TB treatment; this definition included cases diagnosed on the basis of X-ray abnormalities or suggestive histology and extra pulmonary cases without laboratory confirmation.

### 2.3. Data Analysis

We analysed compliance with screening for active TB, LTBI, and the whole protocol, also taking patient characteristics into account. We also assessed the capacity of the adopted procedures to detect LTBI and active TB. In order to do this, we computed odds ratios (ORs) along with their 95% confidence interval (CI) using crude and multivariable logistic regression models. The potential confounders included in multivariable analysis were adjusted for sex, age, presence of cough, education, knowledge of Italian, employment status (employed versus unemployed), years from arrival in Italy and homelessness. We included knowledge of Italian language because it could affect compliance, influencing patient’s ability to understand the motivations of the clinical examinations and therapies proposed. When the use of multivariable analysis led to exceedingly high statistical instability, we limited our assessment to crude estimates only (unadjusted for other variables). Risk analysis was performed by calculating odds ratios estimated from conditional logistic regression with crude and multivariate models. When the odds ratios could not be calculated, we applied the chi2 test and probability according to Fisher’s exact Test. For the evaluation of the differences between continuous variables, we applied the *t*-test. For PE, HS, and IPT, we performed only the crude analysis because of the smallness of the sample, causing too much instability in the statistical analysis.

## 3. Results

From 2012 to 2016, TST was administered to a total number of 404 immigrants. Of these, 36 patients were excluded from our analysis: 8 were born in Italy from undocumented parents and 28 were younger than 15 years. The final study population consisted of 368 immigrants: 186 (50.5%) and 182 (49.5%) were tested in 2012 and 2013, respectively. The demographic characteristics of the study population are reported in Table S1. Adherence to the various stages of the screening procedures and treatment outcomes are shown in Table 1.

Patients who did not return for the reading of TST amounted to 15.2%. Patients who did not undergo CXR, QFT and PE despite the prescriptions amounted to 11.3%, 19.1% and 7.1%, respectively. Finally, 90.2% of subjects completed all steps expected for the screening of active TB, considering TST reading and CXR/PE, when prescribed. In immigrants with a positive TST result, adherence was lower, since their path is usually longer. A total of 5 cases of active TB were diagnosed during the study period. After discharge from hospital, they were followed monthly at TBOA (Outpatient Activity dedicated to management of LTBI and TB at CFF) and they all successfully completed the treatment.

The overall compliance with LTBI screening, which involved TST, CXR, QFT, and PE was 87.3%. Also in this case, adherence of patients with positive TST was observed to be lower (79.9%). LTBI treatment was prescribed to 28 patients, while only 20 out of 28 patients started prophylaxis with Isoniazid, because eight patients had increased levels of transaminases for chronic alcohol abuse or HBsAg, or they refused therapy. Eventually, 14 of the 20 patients who started IPT were able to complete the treatment, whereas 6 patients discontinued therapy voluntarily or after medical indication. HS for 2 years was indicated in 41 patients yet only 6 completed all follow-up. Finally, 76.4% of immigrants completed the whole diagnostic and therapeutic protocol for active TB and LTBI. Considering only TST-positive ones, adherence decreased to 51%.

Table 2 and Table 3 deal with the full path for the screening of active TB and LTBI. In multivariable analysis regarding the screening of active TB (Table 2), it is possible to appreciate that female sex, homelessness and age between 25–34 years were associated with a considerably lower rate of completion of the screening. On the contrary, a higher level of education, time of arrival in Italy for at least 5 years and the presence of cough were all associated with better compliance. With regard to LTBI (Table 3), being a native of the Americas, age between 25 and 34 years, employment, and homelessness were associated with considerably lower adherence. Refugee status, time of arrival in Italy for at least 5 years and the presence of cough were associated with better compliance. It is interesting to note that being from the Americas was associated with very low compliance for both active TB and LTBI screening (OR 0.27, 95% CI 0.05–1.44; OR 0.32, 95% CI 0.06–1.64, respectively). Table 4 outlines compliance with the full algorithm. As can be seen, age had a negative impact on adherence. In addition, being employed, homelessness and prostitution were associated with considerably lower compliance. Being a native of South-East Asia and the Western Pacific (OR 4.48, 95% CI 1.57–12.73), presence of cough, having been in Italy for a longer time, in particular for more than 5 years, and the attainment of higher education levels were observed to be related to a much higher probability of completion. Female Chinese patients’ compliance with the protocol was substantially higher than in the rest of the population (OR 55.63, 95% CI 5.35–578), while Nigerians, Georgians, and Tunisians had lower levels of adhesion (Table S2). 

Considering compliance with TST reading, both bivariate and multivariate analysis show that American and European origin, age between 25–34 years old, prostitution and homelessness were associated with considerably lower rates of return for the reading of the test. On the other hand, higher levels of education were associated with better compliance. In multivariable analysis, the presence of cough was the only symptom associated with greater compliance (OR 7.61, 95% CI 0.92–63.2) (Table S3). Compliance with CXR showed a positive correlation to the presence in Italy for at least 5 years, while a negative correlation was associated to the female sex (Table S4). Compliance with QFT was inversely correlated with education and positively associated with the age group 25–34 years (OR 3.80, 95% CI 0.87–16.53) (Table S5). Female sex, being a native of the Americas, and sufficient or good knowledge of Italian were associated with a 100%-rate of IPT completion. Conversely, refugees showed a tendency to refuse or to voluntarily stop the treatment, and higher education levels inversely correlated with the probability of completion (Table S6).

## 4. Discussion

In our study, adherence with the screening of active TB and LTBI and with pulmonary TB therapy was very high compared to other recent investigations conducted in the USA, Switzerland, and Italy [13,14,15,16,17]. However, a direct comparison between these studies is difficult because study design, countries of origin and sample size were different.

In our study, we identified few fragile groups on which to focus to improve the performance of the screening program [18]. In particular, immigrants from America, Europe, Nigeria, Tunisia, and Georgia showed very low rates of compliance with screening, highlighting the need to target these subgroups with tailored procedures. Immigrants who were employed had lower overall compliance, probably due to the absence of health protection plans and the difficulties in acquiring health permits for these irregular workers. Conversely, knowledge of the Italian language was not significantly associated with compliance. The systematic presence of cultural mediators may have played a key role in determining this outcome, leading the immigrants to fully understand the significance of the screening program and the treatments they underwent, and to freely express questions and concerns [9,19,20]. 

Our study has a few strengths. First of all, the decision to include in the study the whole population of illegal immigrants should be mentioned. Had we not included eligible immigrants from countries with low TB incidence, we would have excluded about 48% of the subjects and missed the 80% of TB cases. Therefore the model adopted in our centre is innovative because it is based on the presence of a stable team of specialists and a nurse dedicated to the periodic monitoring of compliance and to the recovery of the subjects that don’t show up for appointments. Finally, the fact that all the investigations were for free and that there was a direct delivery of the therapy are important elements in ensuring such a high adherence to screening and treatment. 

Our study also has limitation. To begin with, the immigrants’ self-reported data on education, employment status and symptoms are likely to imply an information bias that may have limited the accuracy of such data. Furthermore, we have not studied the correlation between adherence to screening and characteristics such drinking habits and drug abuse, smoking, or possible congenital or acquired immunodeficiency disorders. Moreover, the small size of the study population led to a lack of statistical precision in our results, which need to be confirmed in larger populations. In addition, in patients with negative TST, QFT was not systematically performed and as reported in recent studies [21], QFT might increase the identification of LTBI cases in recent immigrants. Finally, the findings of this study and the investigated setting may not be easily extended to other contexts, since the model adopted in the study centre was based on the fact that all procedures were for free, and were characterized by a stable health care team. This team also included a nurse dedicated to the periodic monitoring of compliance and to making contact with subjects who failed to show up for appointments.

## 5. Conclusions

In conclusion, irregular immigrants appear to be a population with a high prevalence of LTBI and active TB and risk of progression from LTBI to active TB, being also characterized by delays in diagnosis and impaired referral to the health services. Consequently, they should be actively targeted with appropriate screening and follow-up procedures, particularly for selected subgroups showing lower compliance with screening and follow-up. Because of the high probability of diagnostic delay and poor accessibility to health services, systematic active TB screening should be implemented in each clinic dealing with seeking care irregular immigrants. In addition, the simultaneous execution of both the TB and LTBI screenings may optimize allocation and use of the resources.

## Figures and Tables

**Table 1:** Compliance with the different steps of the algorithm and treatment outcomes.

Steps of the Algorithm	*N* Performed/Prescribed	%
**TST read**	349/368	94.8
**CXR performed**	134/151	88.7
**QFT performed**	89/110	80.9
**PE attended**	78/84	92.9
**Screening TB (TST, CXR, PE) completed**	332/368	90.2
negative TST with CXR indication	12/12	100
positive TST	122/139	87.8
**Screening LTBI (TST, CXR, QFT, PE) completed**	317/363	87.3
negative TST with CXR indication	12/12	100
positive TST	107/134	79.9
**LTBI treatment**		
refusal or voluntary interruption	8/28	28.6
medical contraindication or interruption	6/28	21.4
treatment completed	14/28	50.0
**LTBI health surveillance** never started	17/41	41.5
voluntary interruption	18/41	43.9
completed	6/41	14.6
**TB treatment completed**	5/5	100
**Whole algorithm completed**	281/368	76.4
negative TST with CXR indication	12/12	100
positive TST	71/139	51.1

TST: tuberculin skin test; CXR: chest-X-ray; QFT: quantiferon test; PE: pneumological examination; TB: tuberculosis; LTBI: latent tuberculosis infection.

**Table 2:** Association between selected characteristics and compliance with screening for active tuberculosis (TB).

Sample Characteristics	TB Screening Started/Completed		Bivariate OR (95% CI)	*p*-Value	Multivariate OR (95% CI)	*p*-Value
	*N*	%				
**Sex:**						
Male	222/243	91.36	1.00 (reference)		1.00 (reference)	
Female	110/125	88.00	0.69 (0.34–1.40)	0.306	0.47 (0.22–1.03)	0.061
**Age at TST in Years:**						
15–24	90/97	92.78	1.00 (reference)		1.00 (reference)	
25–34	113/130	86.92	0.52 (0.21–1.30)	0.161	0.44 (0.16–1.18)	0.103
35–44	87/95	91.58	0.85 (0.29–2.43)	0.756	0.57 (0.18–1.84)	0.344
≥45	42/46	91.30	0.82 (0.23–2.94)	0.757	0.90 (0.20–4.03)	0.891
Continuous OR			0.99 (0.70–1.41)	0.972	0.96 (0.64–1.43)	0.827
**Region of Origin:**						
Africa	103/111	92.79	1.00 (reference)		1.00 (reference)	
Eastern Mediterranean	75/83	90.36	0.73 (0.26–2.03)	0.544	0.75 (0.25–2.27)	0.607
Europe	60/72	83.33	0.39 (0.15–1.00)	0.051	0.44 (0.15–1.30)	0.138
SE Asia/West Pacific	85/90	94.44	1.32 (0.42–4.19)	0.637	2.49 (.57–10.87)	0.226
Americas	9/12	75.00	0.23 (0.05–1.04)	0.056	0.27 (0.05–1.44)	0.125
**TB Incidence °:**			1.26 (0.89–1.79)	0.187	1.15 (0.79–1.69)	0.458
Any Symptoms *:						
No	211/236	89.41	1.00 (reference)			
Yes	121/132	91.67	1.30 (0.62–2.74)	0.485		
**Cough:**						
No	249/280	88.93	1.00 (reference)		1.00 (reference)	
Yes	83/88	94.32	2.07 (0.78–5.49)	0.145	3.23 (1.04–10.05)	0.043
Education **:			1.36 (0.88–2.12)	0.168	1.49 (0.93–2.40)	0.101
Italian Language **°:			0.89 (0.52–1.50)	0.652	0.90 (0.51–1.58)	0.721
**Employment Status:**						
No	234/258	90.70	1.00 (reference)		1.00 (reference)	
Yes	90/102	88.24	0.77 (0.37–1.60)	0.484	0.79 (0.32–1.96)	0.612
**Years in Italy:**						
<5	223/252	88.49	1.00 (reference)		1.00 (reference)	
≥5	102/109	93.58	1.89 (0.80–4.47)	0.144	3.29 (1.18–9.22)	0.023
Continuous OR			1.06 (0.96–1.18)	0.248	1.18 (0.72–1.92)	0.513
**Homelessness:**						
No	313/345	90.72	1.00 (reference)		1.00 (reference)	
Yes	19/23	82.61	0.49 (0.16–1.52)	0.213	0.35 (0.10–1.25)	0.107
**Refugees:**						
No	304/340	89.41	-			
Yes	28/28	100.00	-	0.070 ***		
**Prostitution:**						
No	323/357	90.48	1.00 (reference)			
Yes	9/11	81.82	0.47 (0.10–2.28)	0.352		
**Pregnancy:**						
No	323/359	89.97	-			
Yes	9/9	100.00	-	0.319 ***		
**TST Result:**						
Negative	210/210	100.00	-			
Positive	122/139	87.77	-	0.000 ***		

°: TB incidence in the country of origin is sub-divided into 4 categories: 0–49, 50–99, 100–199, ≥200 per 100,000 population/year. *: Meaning at least one among cough, hemoptysis, fever, chest pain, weight loss, fatigue, night sweats, chills or loss of appetite. **: Education is sub-divided into three categories: illiterate/primary school, secondary school, high school/degree. **°: Knowledge of Italian is sub-divided into three categories: none, sufficient, good. ***: Two-sample Mann–Whitney test. TB: tuberculosis; LTBI: latent tuberculosis infection; TST: tuberculin skin test; SE Asia: South-East Asia; OR: odds ratio; CI: confidence interval.

**Table 3:** Association between selected characteristics and compliance with screening for latent TB infection (LTBI).

Sample Characteristics	LTBI Screening Started/Completed		Bivariate OR (95% CI)	*p*-Value	Multivariate OR (95% CI)	*p*-Value
	*N*	%				
**Sex:**						
Male	211/241	87.55	1.00 (reference)		1.00 (reference)	
Female	106/122	86.89	0.94 (0.49–1.80)	0.857	0.76 (0.38–1.54)	0.451
**Age at TST in Years:**						
15–24	88/97	90.72	1.00 (reference)		1.00 (reference)	
25–34	107/127	84.25	0.55 (0.24–1.26)	0.157	0.54 (0.22–1.31)	0.173
35–44	80/93	86.02	0.63 (0.26–1.55)	0.314	0.57 (0.21–1.56)	0.273
≥45	42/46	91.30	1.07 (0.31–3.69)	0.910	1.39 (0.33–5.86)	0.658
Continuous OR			0.98 (0.72–1.34)	0.892	1.01 (0.70 –1.45)	0.956
**Region of Origin:**						
Africa	97/109	88.99	1.00 (reference)		1.00 (reference)	
Eastern Mediter.	73/83	87.95	0.90 (0.37–2.20)	0.823	0.93 (0.35–2.47)	0.892
Europe	60/72	83.33	0.62 (0.26–1.47)	0.275	0.72 (0.27–1.91)	0.509
SE Asia/West Pacific	78/87	89.66	1.07 (0.43–2.67)	0.881	2.39 (0.72–7.93)	0.155
Americas	9/12	75.00	0.37 (0.09–1.56)	0.177	0.32 (0.06–1.64)	0.174
**TB Incidence °:**			1.11 (0.82–1.52)	0.489	0.99 (0.70–1.40)	0.975
Any Symptoms *:						
No	203/235	86.38	1.00 (reference)			
Yes	114/128	89.06	1.28 (0.66–2.51)	0.464		
**Cough:**						
No	240/279	86.02	1.00 (reference)		1.00 (reference)	
Yes	77/84	91.67	1.79 (0.77–4.16)	0.178	2.38 (0.92–6.17)	0.073
Education **:			1.09 (0.73–1.63)	0.660	1.09 (0.71–1.68)	0.688
Italian Language **°:			1.13 (0.68–1.87)	0.636	1.15 (0.67–1.95)	0.616
**Employment Status:**						
No	228/257	88.72	1.00 (reference)		1.00 (reference)	
Yes	81/98	82.65	0.61 (0.32–1.16)	0.131	0.56 (0.25–1.26)	0.162
**Years in Italy:**						
<5	215/249	86.35	1.00 (reference)		1.00 (reference)	
≥5	95/107	88.79	1.25 (0.62–2.52)	0.530	1.88 (0.80–4.40)	0.145
Continuous OR			1.04 (0.95–1.13)	0.376	0.99 (0.64–1.54)	0.972
**Homelessness:**						
No	299/340	87.94	1.00 (reference)		1.00 (reference)	
Yes	18/23	78.26	0.49 (0.17–1.40)	0.185	0.35 (0.11–1.12)	0.076
**Refugees:**						
No	290/335	86.57	1.00 (reference)			
Yes	27/28	96.43	4.19 (0.56–31.60)	0.165		
**Prostitution:**						
No	309/352	87.78	1.00 (reference)			
Yes	8/11	72.73	0.37 (0.09–1.45)	0.155		
**Pregnancy:**						
No	309/355	87.04	-			
Yes	8/8	100.00	-	0.277 ***		
**TST result:**						
Negative	210/210	100.00	-			
Positive	107/134	79.85	-	0.000 ***		

°: TB incidence in the country of origin is sub-divided into four categories: 0–49, 50–99, 100–199, ≥200 per 100,000 population/year. *: Meaning at least one among cough, hemoptysis, fever, chest pain, weight loss, fatigue, night sweats, chills or loss of appetite. **: Education is sub-divided into three categories: illiterate/primary school, secondary school, high school/degree. **°: Knowledge of Italian is sub-divided into three categories: none, sufficient, good. ***: Two-sample Mann–Whitney test. TB: tuberculosis; LTBI: latent tuberculosis infection; TST: tuberculin skin test; SE Asia: South-East Asia.

**Table 4:** Association between selected characteristics and compliance with the full protocol (bivariate and multivariate analysis).

Protocol Started/Completed			Bivariate OR (95% CI)	*p*-Value	Multivariate	*p*-Value
Sample characteristics	*N*	%			OR (95% CI)	
**Sex:**						
Male	183/243	75.31	1.00 (reference)		1.00 (reference)	
Female	98/125	78.40	1.19 (0.71–1.99)	0.509	1.18 (0.65–2.12)	0.588
**Age at TST in Years:**						
15–24	83/97	85.57	1.00 (reference)		1.00 (reference)	
25–34	97/130	74.62	0.50 (0.25–0.99)	0.046	0.44 (0.21–0.93)	0.031
35–44	70/95	73.68	0.47 (0.23–0.98)	0.043	0.39 (0.17–0.88)	0.024
≥45	31/46	67.39	0.35 (0.15–0.81)	0.014	0.26 (0.10–0.68)	0.006
Continuous OR			0.74 (0.58–0.94)	0.014	0.67 (0.50–0.89)	0.007
**Region of Origin:**						
Africa	85/111	76.58	1.00 (reference)		1.00 (reference)	
Eastern Mediterranean	62/83	74.70	0.90 (0.47–1.75)	0.763	0.87 (0.41–1.85)	0.723
Europe	49/72	68.06	0.65 (0.34–1.26)	0.205	0.90 (0.42–1.97)	0.801
SE Asia/West Pacific	76/90	84.44	1.66 (0.81–3.41)	0.167	4.48 (1.57–12.73)	0.005
Americas	9/12	75.00	0.92 (0.23–3.64)	0.903	0.67 (0.14–3.16)	0.617
**TB Incidence °:**			0.91 (0.72–1.16)	0.461	0.79 (0.59–1.05)	0.107
Any Symptoms *:						
No	179/236	75.85	1.00 (reference)			
Yes	102/132	77.27	1.08 (0.65–1.79)	0.758		
**Cough:**						
No	212/280	75.71	1.00 (reference)		1.00 (reference)	
Yes	69/88	78.41	1.16 (0.65–2.07)	0.604	1.75 (0.88–3.46)	0.110
Education **:			1.18 (0.86–1.62)	0.306	1.24 (0.88–1.74)	0.220
Italian Language **°:			1.26 (0.85–1.89)	0.254	1.30 (0.82–2.04)	0.265
**Employment Status:**						
No	201/258	77.91	1.00 (reference)		1.00 (reference)	
Yes	75/102	73.53	0.79 (0.46–1.34)	0.377	0.59 (0.30–1.17)	0.133
**Years in Italy:**						
<5	188/252	74.60	1.00 (reference)		1.00 (reference)	
≥5	88/109	80.73	1.43 (0.82–2.48)	0.209	2.94 (1.44–6.01)	0.003
Continuous OR			1.06 (0.99–1.13)	0.119	1.30 (0.90–1.86)	0.160
**Homelessness:**						
No	267/345	77.39	1.00 (reference)		1.00 (reference)	
Yes	14/23	60.87	0.45 (0.19–1.09)	0.077	0.35 (0.13–0.96)	0.042
**Refugees:**						
No	258/340	75.88	1.00 (reference)			
Yes	23/28	82.14	1.46 (0.54–3.97)	0.456		
**Prostitution:**						
No	273/357	76.47	1.00 (reference)			
Yes	8/11	72.73	0.82 (0.21–3.16)	0.774		
**Pregnancy:**						
No	272/359	75.84	-			
Yes	9/9	91.67	-	0.091 ***		
**TST Result:**						
Negative	210/210	100.00	-			
Positive	71/139	51.08	-	0.000 ***		

° TB incidence in the country of origin is sub-divided into 4 categories: 0–49, 50–99, 100–199, ≥200 per 100,000 population/year. * Meaning at least one among cough, hemoptysis, fever, chest pain, weight loss, fatigue, night sweats, chills or loss of appetite. ** Education is sub-divided into three categories: illiterate/primary school, secondary school, high school/degree. **° Knowledge of Italian is sub-divided into three categories: none, sufficient, good. *** Two-sample Mann–Whitney test. TST: tuberculin skin test; SE Asia: South-East Asia; TB: tuberculosis.

## Supplementary Materials

The following are available online at http://www.mdpi.com/1660-4601/16/1/28/s1, Table S1: Demographic characteristics of the study population. Table S2: Association between countries of origin, sex and compliance with screening for active tuberculosis (TB), screening for latent tuberculosis infection (LTBI) and the full protocol (multivariate analysis). Table S3: Association between selected characteristics and compliance with Tuberculin Skin Test (TST) reading (bivariate and multivariate analysis). Table S4: Association between selected characteristics and compliance with chest-X-ray (CXR) execution (bivariate and multivariate analysis). Table S5: Association between selected characteristics and compliance with Quantiferon-TB gold (QFT) execution (bivariate and multivariate analysis). Table S6: Association between selected characteristics and outcome of Latent Tuberculosis Infection (LTBI) treatment (bivariate analysis).
