# Supplementary material for: Compliance with Tuberculosis Screening in Irregular Immigrants
Source: Int J Environ Res Public Health. 2018 Dec 23;16(1):28. doi: 10.3390/ijerph16010028 (PMC6339090; doi:10.3390/ijerph16010028)
Supplement: Supplementary file 1 [file ijerph-16-00028-s001.pdf]

SUPPLEMENTARY MATERIAL

Table S1. Demographic characteristics of the study population (TOT = 368).

	N	%
Sex:		
Male	243	66.03
Female	125	33.97
Age in years:		
15-24	97	26.36
25-34	130	35.33
35-44	95	25.82
≥ 45	46	12.50
Region of origin*:		
African	111	30.16
Eastern Mediterranean	83	22.55
European	72	19.57
South-East Asia	4	1.09
Western Pacific	86	23.37
Americas	12	3.26
Countries with > 5% population:		
China	86	23.37
Nigeria	63	17.12
Pakistan	28	7.61
Georgia	26	7.07
Tunisia	24	6.52
Morocco	22	5.98
TB incidence in the country of origin**:		
0-49	46	12.50
50-99	132	35.87
100-199	96	25.27
≥ 200	97	26.36
Living with family:		
No	261	70.92
Yes	94	25.54
Unknown	13	3.53
N° of cohabitants:		
0-2	146	39.67
3-4	104	28.26
≥ 5	45	12.23
Unknown	73	19.84
Any symptoms of active TB:***	132	35.87
cough > 2 weeks	37	10.05
cough ≥ 2 weeks	51	13.86
hemoptysis	8	2.17
fever	40	10.87
chest pain	5	1.36
weight loss	10	2.72
fatigue	23	6.25
night sweats	7	1.90
chills	11	2.99
loss of appetite	15	4.08
Peripheral lymphadenopathy:	14	3.80

Any symptoms and/or peripheral lymphadenopathy:***	144	39.13
Years from arrival in Italy at TST:		
< 1	121	32.88
[1, 2)	49	13.32
[2, 5)	82	22.28
≥5	109	29.62
Unknown	7	1.90
Education:		
Illiterate/Primary school	77	20.92
Secondary school	133	36.14
High school/Degree	147	39.95
Unknown	11	2.99
Knowledge of Italian language:		
None	233	63.32
Sufficient	99	26.90
Good	30	8.15
Unknown	6	1.63
Employment Status:		
No	258	70.11
Yes	102	27.72
Unknown	8	2.17
BCG vaccination:		
No	17	4.62
Yes	78	21.20
Unknown	273	74.18
Lifestyle and other risk factors for TB and LTBI:		
Homeless	23	6.25
Refugees	28	7.61
Transsexual	6	1.63
Prostitution	11	2.99
Pregnancy	9	2.45
Cigarette smokers (≥ 10/die)	48	13.04
Alcohol consumption	27	7.34
Drugs consumption	12	3.26
Stay in prison	5	1.36
Diabetes	7	1.90
BPCO	3	0.82
HIV	1	0.27
Other causes of immunosuppression	3	0.82
Other infections:		
HBV	7	1.90
HCV	7	1.90
LUES	6	1.63

*Countries of origin are grouped according to WHO's Regions. ** Number of cases of active TB per 100,000 population/year - Data from the WHO Global TB Report 2015 (population at 2014). *** Meaning at least one among cough, hemoptysis, fever, chest pain, weight loss, fatigue, night sweats, chills or loss of appetite. **TB:** tuberculosis; **LTBI:** latent tuberculosis infection; **BCG:** bacillus Calmette–Guérin; **COPD:** chronic obstructive pulmonary disease; **HIV:** human immunodeficiency virus; **HBV**: Hepatitis B virus; **HCV:** hepatitis C virus.

Table S2. Association between countries of origin, sex and compliance with screening for active tuberculosis (TB), screening for latent tuberculosis infection (LTBI) and the full protocol (multivariate analysis*).

	TB screening started/completed		LTBI screening started/completed		Protocol started/completed	
	**OR (95% CI)**	**P**	**OR (95% CI)**	**P**	**OR (95% CI)**	**P**
**China**	10.37 (2.32-46.38)	0.002	4.98 (1.52-16.33)	0.008	6.68 (2.35-19.04)	0.000
Males	12.12 (1.08-136.21)	0.043	3.99 (0.85-18.65)	0.079	3.43 (0.96-12.22)	0.058
Females	19.20 (1.76-209.36)	0.015	21.08 (1.88-236.4)	0.013	55.63 (5.35-578)	0.001
**Nigeria**	0.62 (0.17-2.33)	0.482	0.43 (0.14-1.36)	0.151	0.48 (0.20-1.15)	0.098
Males	1.32 (0.13-13.37)	0.817	0.51 (0.11-2.25)	0.374	0.39 (0.14-1.10)	0.075
Females	1.24 (0.10-14.74)	0.864	1.01 (0.09-11.82)	0.992	2.07 (0.27-15.72)	0.480
**Pakistan**	2.17 (0.24-19.22)	0.487	0.90 (0.22-3.78)	0.891	0.92 (0.32-2.66)	0.883
**Georgia**	0.12 (0.03-0.42)	0.001	0.23 (0.07-0.73)	0.012	0.46 (0.18-1.21)	0.117
Males	0.07 (0.01-0.45)	0.005	0.22 (0.04-1.09)	0.064	0.42 (0.11-1.55)	0.192
Females	0.12 (0.02-0.80)	0.029	0.14 (0.02-0.89)	0.037	0.50 (0.11-2.32)	0.373
**Tunisia**	0.29 (0.06-1.32)	0.109	0.23 (0.06-0.94)	0.041	0.20 (0.06-0.71)	0.013
Males	0.36 (0.06-2.05)	0.252	0.18 (0.04-0.93)	0.040	0.22 (0.06-0.87)	0.030
Females	-	-	-	-	-	-
**Morocco**	1.14 (0.21-6.13)	0.882	4.25 (0.48-37.96)	0.195	1.49 (0.42-5.31)	0.536

* Adjusted for sex, age, TB incidence in the country of origin, presence of cough, education, knowledge of Italian, employment status and homelessness. **TB:** tuberculosis; **LTBI**: latent tuberculosis infection.

Table S3. Association between selected characteristics and compliance with Tuberculin Skin Test (TST) reading (bivariate and multivariate analysis).

	TST read/executed		Bivariate		Multivariate	
	**N**	**%**	**OR (95% CI)**	***p value***	**OR (95% CI)**	**P**
Sex:						
Male	233/243	95.88	1.00 (referent)		1.00 (referent)	
Female	116/125	92.80	0.55 (0.22-1.40)	0.211	0.26 (0.09-0.79)	0.017
Age at TST in years:						
15-24	94/97	96.91	1.00 (referent)		1.00 (referent)	
25-34	119/130	91.54	0.34 (0.09-1.27)	0.110	0.21 (0.06-0.68)	0.009

35-44	91/95	95.79	0.73 (0.16-3.33)	0.681	0.31 (0.05-1.80)	0.192
≥45	45/46	97.83	1.44 (0.14-14.19)	0.757	-	
Continuous OR			1.10 (0.68-1.77)	0.693	1.07 (0.61-1.88)	0.804
Region of origin:						
Africa	108/114	97.30	1.00 (referent)		1.00 (referent)	
Eastern Mediter.	78/83	93.98	0.43 (0.10-1.87)	0.262	0.41 (0.08-2.09)	0.281
Europe	66/72	91.67	0.31 (0.07-1.26)	0.102	0.35 (0.06-1.86)	0.216
SE Asia/West Pacif.	87/90	96.67	0.81 (0.16-4.09)	0.794	2.14 (0.26-17.92)	0.483
Americas	10/12	83.33	0.14 (0.02-0.93)	0.042	0.11 (0.01-0.99)	0.049
TB incidence°:			1.53 (0.94-2.47)	0.085	1.39 (0.82-2.36)	0.226
Any symptoms*:						
No	222/236	94.07	1.00 (referent)			
Yes	127/132	96.21	1.60 (0.56-4.55)	0.377		
Cough:						
No	263/280	93.93	1.00 (referent)		1.00 (referent)	
Yes	86/88	97.73	2.78 (0.63-12.27)	0.177	7.61 (0.92-63.2)	0.060
Education**:			1.87 (1.02-3.39)	0.044	2.20 (1.11-4.35)	0.023
Italian language**°:			0.99 (0.47-2.06)	0.972	1.06 (0.48-2.37)	0.884
Employment status:						
No	246/258	95.35	1.00 (referent)		1.00 (referent)	
Yes	95/102	93.14	0.66 (0.25-1.73)	0.401	0.71 (0.21-2.41)	0.577
Years in Italy:						
< 5	237/252	94.05	1.00 (referent)		1.00 (referent)	
≥5	105/109	96.33	1.66 (0.54-5.13)	0.377	3.02 (0.74-12.38)	0.124
Continuous OR			1.08 (0.93-1.25)	0.325	1.37 (0.69-2.72)^	0.364
Homelessness:						

No	329/345	95.36	1.00 (referent)		1.00 (referent)	
Yes	20/23	86.96	0.32 (0.09-1.20)	0.093	0.17 (0.04-0.83)	0.029
Refugees:						
No	321/340	94.41	-			
Yes	28/28	100.00	-	0.200***		
Prostitution:						
No	340/357	95.24	1.00 (referent)			
Yes	9/11	81.82	0.22 (0.04-1.12)	0.069		
Pregnancy:						
No	340/359	94.71	-			
Yes	9/9	100.00	-	0.479***		

° TB incidence in the country of origin is sub-divided into 4 categories: 0-49, 50-99, 100-199, ≥200 per 100,000 population/year. * Meaning at least one among cough, hemoptysis, fever, chest pain, weight loss, fatigue, night sweats, chills or loss of appetite. ** Education is sub-divided into three categories: Illiterate/Primary school, Secondary school, High school/Degree. **° Knowledge of Italian is sub-divided into three categories: None, Sufficient, Good. *** Two-sample Mann-Whitney test. **TST**: tuberculin skin test; **SE Asia**: South-East Asia**; TB:** tuberculosis.

Table S4. Association between selected characteristics and compliance with Chest-X-Ray (CXR) execution (bivariate and multivariate analysis).

	CXR performed/ prescribed		Bivariate		Multivariate	
	N	%	OR (95% CI)	P	OR (95% CI)	P
Sex:						
Male	99/110	90.00	1.00 (referent)		1.00 (referent)	
Female	35/41	85.37	0.65 (0.22-1.88)	0.426	0.40 (0.12-1.37	0.145
Age at TST in years:						
15-24	21/25	84.00	1.00 (referent)		1.00 (referent)	
25-34	45/51	88.24	1.43 (0.36-5.60)	0.609	1.38 (0.31-6.17)	0.675
35-44	48/52	92.31	2.29 (0.52-10.02)	0.273	1.86 (0.36-9.56)	0.455

≥45	20/23	86.96	1.27 (0.25-6.40)	0.772	1.33 (0.23-7.81)	0.755
Continuous OR			1.18 (0.69-2.03)	0.545	1.14 (0.64-2.04)	0.655
Region of origin:						
Africa	45/50	90.00	1.00 (referent)		1.00 (referent)	
Eastern Mediter.	25/28	89.29	0.93 (0 .20-4.20)	0.921	0.71 (0.14-3.56)	0.680
Europe	25/31	80.65	0.46 (0.13-1.67)	0.240	0.41 (0.09-1.89)	0.252
SE Asia/West Pacif.	37/39	94.87	2.05 (0.38- 11.22)	0.405	1.85 (0.18-19.37)	0.608
Americas	2/3	66.67	0.22 (0.02-2.9)	0.252	0.20 (0.01-6.16)	0.360
TB incidence °:			1.11 (0.66-1.88)	0.689	1.39 (0.70-2.76)	0.339
Any symptoms*:						
No	79/90	87.78	1.00 (referent)			
Yes	55/61	90.16	1.28 (0.44-3.66)	0.650		
Cough:						
No	96/110	87.27	1.00 (referent)		1.00 (referent)	
Yes	38/41	92.68	1.85 (0.50-6.79)	0.356	2.21 (0.52-9.37)	0.284
Education**:			0.87 (0.45-1.70)	0.686	0.96 (0.47-1.95)	0.902
Italian language**°:			0.70 (0.34-1.46)	0.345	0.67 (0.31-1.47)	0.318
Employment status:						
No	85/97	87.63	1.00 (referent)		1.00 (referent)	
Yes	44/49	89.80	1.24 (0.41-3.75)	0.700	1.70 (0.37-7.79)	0.494
Years in Italy:						
< 5	79/93	84.95	1.00 (referent)		1.00 (referent)	
≥ 5	50/53	94.34	2.95 (0.81-10.80)	0.102	2.76 (0.65-11.65)	0.168
Continuous OR			1.10 (0.93-1.29)	0.259	1.09 (0.54-2.21)^	0.814
Homelessness:						
No	123/139	88.49	1.00 (referent)		1.00 (referent)	

Yes	11/12	91.67	1.43 (0.17-11.83)	0.740	1.46 (0.13-16.20)	0.757
Refugees:						
No	126/143	88.11	-			
Yes	8/8	100.00	-	0.302***		
Prostitution:						
No	133/150	88.67	-			
Yes	1/1	100.00	-	0.722***		
Pregnancy:						
No	132/149	88.59	-			
Yes	5/5	100.00	-	0.613***		

°TB incidence in the country of origin is sub-divided into 4 categories: 0-49, 50-99, 100-199, ≥200 per 100,000 population/year. * Meaning at least one among cough, hemoptysis, fever, chest pain, weight loss, fatigue, night sweats, chills or loss of appetite. ** Education is sub-divided into three categories: Illiterate/Primary school, Secondary school, High school/Degree. **° Knowledge of Italian is sub-divided into three categories: None, Sufficient, Good. *** Two-sample Mann-Whitney test. **CXR:** chest-X-ray; **TST**: tuberculin skin test; **SE-Asia:** South-East Asia; **TB**: tuberculosis.

Table S5. Association between selected characteristics and compliance with Quantiferon-TB gold (QFT) execution (bivariate and multivariate analysis).

	QFT performed/ prescribed		Bivariate		Multivariate	
	N	%	OR (95% CI)	P	OR (95% CI)	P
Sex:						
Male	71/87	81.61	1.00 (referent)		1.00 (referent)	
Female	18/23	78.26	0.81 (0.26-2.51)	0.717	0.65 (0.19-2.23)	0.490
Age at TST in years:						
15-24	14/20	70.00	1.00 (referent)		1.00 (referent)	
25-34	39/46	84.78	2.39 (0.68-8.33)	0.172	3.80 (0.87-16.53)	0.076
35-44	32/39	82.05	1.96 (0.56-6.90)	0.295	3.23 (0.63-16.42)	0.158

≥45	4/ 5	80.00	1.71 (0.16-18.73)	0.659	1.76 (0.12-25.18)	0.679
Continuous OR			1.26 (0.69-2.29)	0.446	1.43 (0.67-3.04)	0.350
Region of origin:						
Africa	33/40	82.50	1.00 (referent)		1.00 (referent)	
Eastern Mediter.	19/23	82.61	1.01 (0.26-3.89)	0.991	0.64 (0.13-3.18)	0.582
Europe	10/15	66.67	0.42 (0.11-1.63)	0.213	0.44 (0.08-2.34)	0.333
SE Asia/West Pacif.	25/29	86.21	1.33 (0.35-5.03)	0.679	5.73 (0.48-69.12)	0.169
Americas	2/3	66.67	0.42 (0.03-5.35)	0.507	0.02 (0.00-0.80)	0.039
TB incidenceº:			1.01 (0.62-1.64)	0.973	1.18 (0.58-2.41)	0.653
Any symptoms*:						
No	60/75	80.00	1.00 (referent)			
Yes	29/35	82.86	1.21 (0.42-3.44)	0.723		
Cough:						
No	73/90	81.11	1.00 (referent)		1.00 (referent)	
Yes	16/20	80.00	0.93 (0.28-3.14)	0.909	0.71 (0.18-2.79)	0.620
Education**:			0.44 (0.22-0.89)	0.023	0.35 (0.15-0.81)	0.014
Italian language**º:			1.23 (0.56-2.73)	0.605	1.42 (0.60-3.35)	0.420
Employment status:						
No	55/69	79.71	1.00 (referent)		1.00 (referent)	
Yes	29/36	80.56	1.05 (0.38-2.90)	0.918	0.61 (0.12-3.01)	0.543
Years in Italy:						
< 5	51/65	78.46	1.00 (referent)		1.00 (referent)	
≥5	33/40	82.50	1.29 (0.47-3.54)	0.616	1.37 (0.37-5.04)	0.640
Continuous OR			1.07 (0.92-1.25)	0.370	1.15 (0.56-2.38)	0.701
Homelessness:						
No	84/103	81.55	1.00 (referent)		1.00 (referent)	

Yes	5/7	71.43	0.57 (0.10-3.14)	0.514	0.24 (0.03-2.05)	0.191
Refugees:						
No	82/102	80.39	1.00 (referent)			
Yes	7/8	87.50	1.71 (0.20-14.68)	0.626		
Prostitution:						
No	89/109	81.65	-			
Yes	0/1	0.00	-	0.039***		
Pregnancy:						
No	88/109	80.73	-			
Yes	1/1	100.00	-	0.627***		

° TB incidence in the country of origin is sub-divided into 4 categories: 0-49, 50-99, 100-199, ≥200 per 100,000 population/year. * Meaning at least one among cough, hemoptysis, fever, chest pain, weight loss, fatigue, night sweats, chills or loss of appetite. ** Education is sub-divided into three categories: Illiterate/Primary school, Secondary school, High school/Degree. **° Knowledge of Italian is sub-divided into three categories: None, Sufficient, Good. *** Two-sample Mann-Whitney test. **QFT:** quantiferon-TB gold; **TST**: tuberculin skin test; **SE Asia**: South-East Asia; **TB:** tuberculosis.

Table S6. Association between selected characteristics and outcome of Latent Tuberculosis Infection (LTBI) treatment (bivariate analysis).

	Refusal or voluntary interruption		Medical contraindication or interruption		Treatment completed		P
	N	%	N	%	N	%	
Sex:							
Male	8/24	33.33	6/24	25.00	10/24	41.67	
Female	0/14	0.00	0/14	0.00	4/24	100.00	0.150
Age at TST in years:							
15-35	7/23	30.43	5/23	21.74	11/23	47.83	
≥36	1/5	20.00	1/5	20.00	3/5	60.00	1.000
Region:							
African	4/14	28.57	3/14	21.43	7/14	50.00	
Eastern Mediterranean	3/6	50.00	0/6	0.00	3/6	50.00	
European	-	-	-	-	-	-	
SE Asia/West Pacif	1/7	14.29	3/7	42.86	3/7	42.86	
Americas	0/1	0.00	0/1	0.00	0/1	100.00	0.578
TB incidence:							
0-99	1/10	10.00	4/10	40.00	5/10	50.00	
≥100	7/18	38.89	2/18	11.11	9/18	50.00	0.129
Any symptoms of active TB:*							
No	7/20	35.00	3/20	15.00	10/20	50.00	

Yes	1/8	12.50	3/8	37.50	4/8	50.00	0.407
Cough:							
No	8/26	30.77	5/26	19.23	13/26	50.00	
Yes	0/2	0.00	0/2	50.00	1/2	50.00	0.463
Education:							
Illiterate/Primary school	2/8	25.00	0/8	0.00	6/8	75.00	
Secondary school	1/8	12.50	2/8	25.00	5/8	62.50	
High school/Degree	4/10	40.00	4/10	40.00	2/10	20.00	0.106
Knowledge of Italian:							
None	8/23	34.78	6/23	26.09	9/23	39.13	
Sufficient	0/3	0.00	0/3	0.00	3/3	100.00	
Good	0/2	0.00	0/2	0.00	2/2	100.00	0.302
Employment status:							
No	6/20	30.00	3/20	15.00	11/20	55.00	
Yes	1/7	14.29	3/7	42.86	3/7	42.86	0.430
Years from arrival in Italy:							
< 5	6/18	33.33	3/18	16.67	9/18	50.00	
≥5	1/8	12.50	3/8	37.50	4/8	50.00	0.396
Alcohol consumption:							
No	8/24	30.77	4/26	15.38	14/26	53.85	
Yes	0/2	0.00	2/2	100.00	0/2	0.00	0.040
Refugees:							
No	5/23	21.74	6/23	26.09	12/23	52.17	
Yes	3/5	60.00	0/5	0.00	2/5	40.00	0.194

*Meaning at least one among cough, hemoptysis, fever, chest pain, weight loss, fatigue, night sweats, chills or loss of appetite. **TST:** tuberculosis skin test; **SE Asia:** South-East Asia; **TB:** tuberculosis.
